# Shear Stress Regulates Osteogenic Differentiation of Human Dental Pulp Stem Cells via the p38 Pathway

**DOI:** 10.3390/ijms26125667

**Published:** 2025-06-13

**Authors:** Hnin Yu Lwin, Watcharaphol Tiskratok, Maythwe Kyawsoewin, Jeeranan Manokawinchoke, Chutimon Termkwanchareon, Nuttapol Limjeerajarus, Chalida Nakalekha Limjeerajarus, Hiroshi Egusa, Thanaphum Osathanon, Phoonsuk Limraksasin

**Affiliations:** 1 Center of Excellence for Dental Stem Cell Biology, Faculty of Dentistry, Chulalongkorn University, Bangkok 10330, Thailand; hnin.y@chula.ac.th (H.Y.L.); leomaythwe@gmail.com (M.K.); jeeranan.m@chula.ac.th (J.M.); chutimon.mon1347@gmail.com (C.T.); thanaphum.o@chula.ac.th (T.O.); 2 Department of Anatomy, Faculty of Dentistry, Chulalongkorn University, Bangkok 10330, Thailand; 3 School of Geriatric Oral Health, Institute of Dentistry, Suranaree University of Technology, Nakhon Ratchasima 30000, Thailand; 4 Office of Academic Affairs, Faculty of Dentistry, Chulalongkorn University, Bangkok 10330, Thailand; nuttapol.l@chula.ac.th; 5 Department of Physiology, Faculty of Dentistry, Chulalongkorn University, Bangkok 10330, Thailand; chalida.n@chula.ac.th; 6 Division of Molecular and Regenerative Prosthodontics, Tohoku University Graduate School of Dentistry, Sendai 980-8575, Miyagi, Japan; 7 Center for Advanced Stem Cell and Regenerative Research, Tohoku University Graduate School of Dentistry, Sendai 980-8575, Miyagi, Japan

**Keywords:** human dental pulp stem cells, shear stress, osteogenesis

## Abstract

This study aimed to investigate the effects of shear stress on osteogenic differentiation of human dental pulp stem cells (hDPSCs). The hDPSCs were subjected to shear stress for 24 h before osteogenic induction for 21 days. The mRNA expression of osteogenic markers such as RUNX2, OSX, ALP, COL1A1, OCN, and OPN was evaluated by real-time RT-PCR. Alkaline Phosphatase (ALP) activity and Alizarin Red S (ARS) staining were investigated to confirm osteogenic differentiation and mineralization of hDPSCs, respectively. The protein expression of osterix was shown by immunofluorescence staining and Western blotting. RNA sequencing was performed to investigate how shear stress affects the osteogenic differentiation of hDPSCs, which was validated through p38 inhibitor (SB203580) treatment. Real-time RT-PCR revealed that shear stress enhanced osteogenic marker-gene expression. The increased osterix protein expression was detected on Day 14 in the shear-stress loading group compared to the static group. Shear stress enhanced ALP activity and mineralization, observed on Days 14 and 21. A volcano plot exhibited up- and downregulated genes, while the p38 inhibitor markedly inhibited osteogenic differentiation of hDPSCs triggered by shear stress. In conclusion, shear stress promotes the osteogenic differentiation of hDPSCs through the p38 mitogen-activated protein kinase signaling pathway.

## 1. Introduction

Regenerative bone therapy is now advanced and popular in dentistry for treatment related to implant placement and aesthetic concerns. Alveolar ridge resorption following tooth extraction often renders lateral bone augmentation inevitable; however, some patients suffer from severe graft resorption during graft healing and follow-up. Although several methods have been developed to overcome critical-size defect healing, there are still limitations regarding individual conditions. For instance, a previous report showed that individual phenotypic dimensions of the bony envelope affect buccal bone healing when applying a guided bone-regeneration concept [1]. Therefore, regenerative bone therapy still awaits effective methods to guide efficient bone repair. Despite autologous grafts being the gold standard, stem cell-based tissue engineering is considered as a powerful alternative for alveolar bone augmentation.

Mesenchymal stem/stromal cells (MSCs) possess heterogeneous properties of self-renewal and differentiation into specific functional cell types such as osteoblasts, adipocytes or fibroblasts [2]. MSCs reside in multiple tissues such as bone marrow, adipose tissue [3], cartilage [4], umbilical cord [5], placenta [6], and stem cells found in dental tissues [7]. Dental pulp stem cells (DPSCs) can be harvested from the pulp tissue of permanent teeth. DPSCs possess the MSCs’ phenotype, and have been reported to differentiate into neurons, chondrocytes, osteoblasts, and liver cells [8]. Thus, human dental pulp stem cells (hDPSCs) may become the alternative noninvasive source for regenerative therapy, as they possess multipotency and can be harvested from extracted impacted third molars and supernumerary teeth [9]. Many approaches have been focused on their dentine- and bone-forming properties [10,11]; thus, the hDPSCs are expected to be a promising source for efficient alveolar bone augmentation via stem cell-based regenerative therapy. The mechanical stimuli are expected to facilitate osteogenic differentiation, as reported in several previous studies, to promote the osteogenic differentiation of stem cells [12,13]. A report demonstrated the effects of shear stress on osteoblast-like cells derived from hDPSCs, and suggested that hDPSCs, like osteogenic cells, acquire responsiveness to pulsating fluid shear stress in mineralizing conditions [14].

The hDPSCs are highly responsive to mechanical stimuli which can modulate their proliferation and differentiation, particularly toward osteogenic lineages. Low-intensity pulsed ultrasound (LIPUS) can induce the proliferation of hDPSCs. Mechanosensitive channels like Piezo-1/2 protein are mediated via ERK 1/2 MAPK signaling pathways [15]. Cyclic mechanical tension increased the proliferation and mRNA expression levels of the osteogenic markers [16], and thus can be considered a potent positive modulator of osteogenic differentiation by hDPSCs. Other mechanical stimuli such as pulsating fluid flow (PFF), static tensile strain, and micro/nanotopographies support the osteogenicity of hDPSCs. These stimuli elevate levels of nitric oxide (NO), PGE2, and COX-2, all associated with enhanced mechanosensitivity and osteogenic activity. In vitro mechanical stimuli including PFF, equiaxial static tensile strain and micro- and nano-scale surface topographies have also been shown to induce osteogenic differentiation of hDPSCs [17,18,19]. Previous studies showed that shear stress enhanced the osteogenic differentiation of MSCs [20], osteoblasts [21], and osteocytes [22] in different manners, depending on the magnitude, duration, and frequency of the applied shear stress; however, the study on the mechanism of osteogenesis differentiation of shear stress-induced hDPSCs is unclear. This may necessitate the optimization of shear stress magnitude to promote osteogenic differentiation of hDPSCs effectively.

Shear stress promotes osteogenic differentiation of stem cells; however, its role in osteogenic differentiation of hDPSCs has not yet been clarified. Therefore, we hypothesized that specific shear stress could enhance the osteogenic differentiation of hDPSCs. The objectives are to study the effects of shear stress on osteogenic differentiation of hDPSCs and clarify the underlying mechanisms. The application of this shear stress to improve tissue-engineered bone and to understand the mechanisms from hDPSCs could bring about technological innovation in regenerative dentistry research and have the potential to restore bone height and width for prosthetic dental/implant treatment.

## 2. Results

### 2.1. Shear Stress Induced Osteogenic Differentiation of hDPSCs

**Figure 1:**
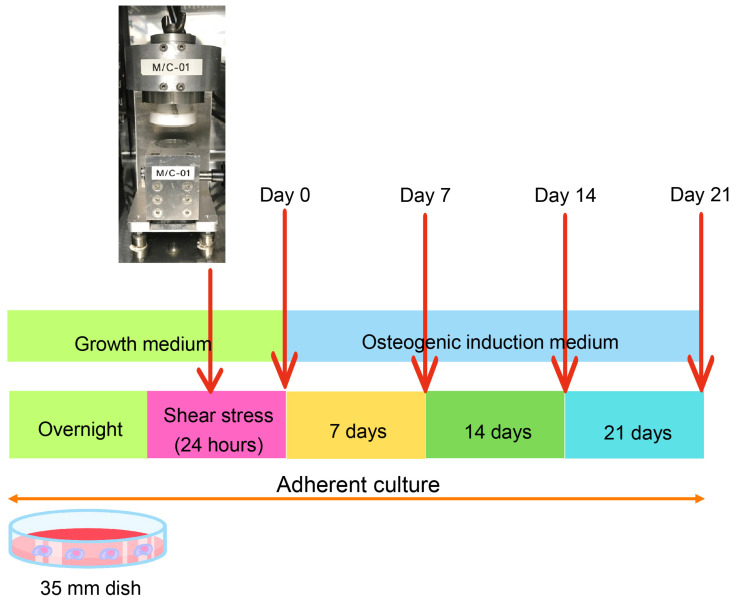
The schematic diagram of the procedure of the present study.

**Figure 2:**
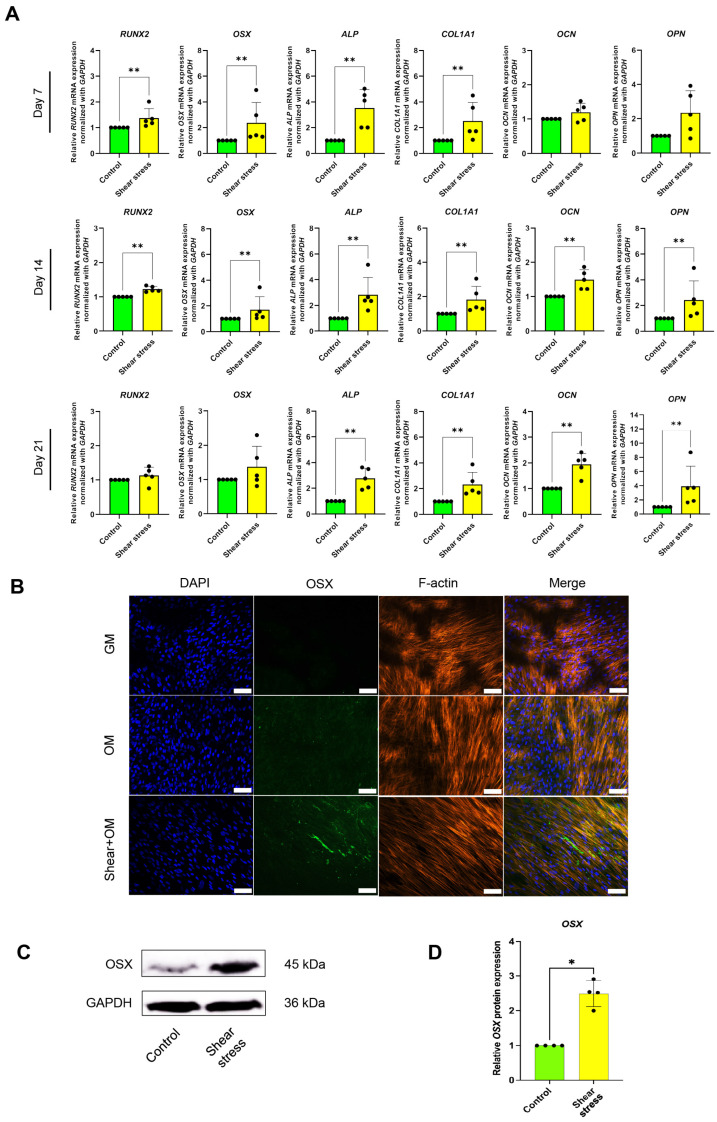
Shear-stress application promoted osteogenic differentiation of human dental pulp stem cells (hDPSCs). (**A**) Real-time reverse-transcription–polymerase chain reaction (RT-PCR) was performed to detect mRNA expression levels of osteogenic marker genes, including *RUNX2*, *OSX*, *ALP*, *COL1A1*, *OCN*, and *OPN*. The expression of *GAPDH* was used as an internal control. Data were statistically analyzed by Mann–Whitney U test. (The present study included data from five different donors: *n* = 5, *p* * < 0.05, ** < 0.001). (**B**) Immunofluorescence analysis was performed to detect the protein expression and localization of OSX (green). hDPSCs cultured under a normal growth medium (GM) were used as a negative control. The cytoskeleton (F-actin; red) and nuclei (blue) were stained using rhodamine–phalloidin and DAPI, respectively. Scale bars: 50 μm. (**C**) Western blot analysis evaluated OSX protein expression on Day 14 of osteogenic differentiation. (**D**) Quantitative analysis of OSX expression, using ImageJ software (version 1.54g). The expression levels were normalized to GAPDH expression. Data were statistically analyzed by Mann–Whitney U test. (*n* = 4: different donors, *p* * < 0.05, ** < 0.001).

After 24 h shear-stress application, the hDPSCs were cultured in an osteogenic media (OM) for an additional 21 days to assess the role of shear stress in the osteogenic differentiation of hDPSCs (Figure 1). Real-time PCR analysis showed that shear stress significantly enhanced the early osteogenic marker gene expression, including the Runt-related transcription factor 2 (*RUNX2*), osterix (*OSX*), alkaline phosphatase (*ALP*), and Type I collagen (*COL1A1*), by 7 days osteogenic differentiation incubation (Figure 2A). The significant upregulation of all osteogenic markers, such as *OSX*, *ALP*, *COL1A1*, Osteocalcin (*OCN*), and Osteopontin (*OPN*), by shear stress, was observed on Day 14 (Figure 2A). After 21-day incubation, shear stress significantly enhanced the expression of *ALP*, *COL1A1*, *OCN*, and *OPN* compared to the control static culture group (Figure 2A). We further examined the osterix (OSX) protein expression by immunofluorescence staining and Western blotting. OSX expression was observed by Day 14 in the static culture group under OM (Figure 2B). The expression of OSX was further enhanced in the shear-stress loading group compared to the static group under OM (Figure 2B). No OSX expression was detected in the static culture under a normal growth medium (GM) (Figure 2B). A significant increase in OSX expression in the shear stress group was shown by Day 14 under OM, compared to the control group (Figure 2B–D).

### 2.2. Shear Stress Promoted Mineralization of Osteogenically Induced hDPSCs

**Figure 3:**
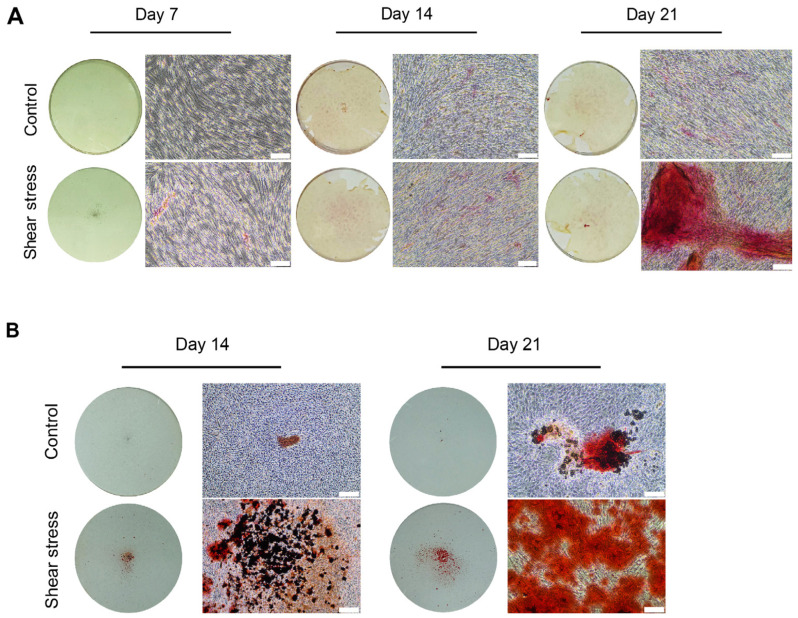

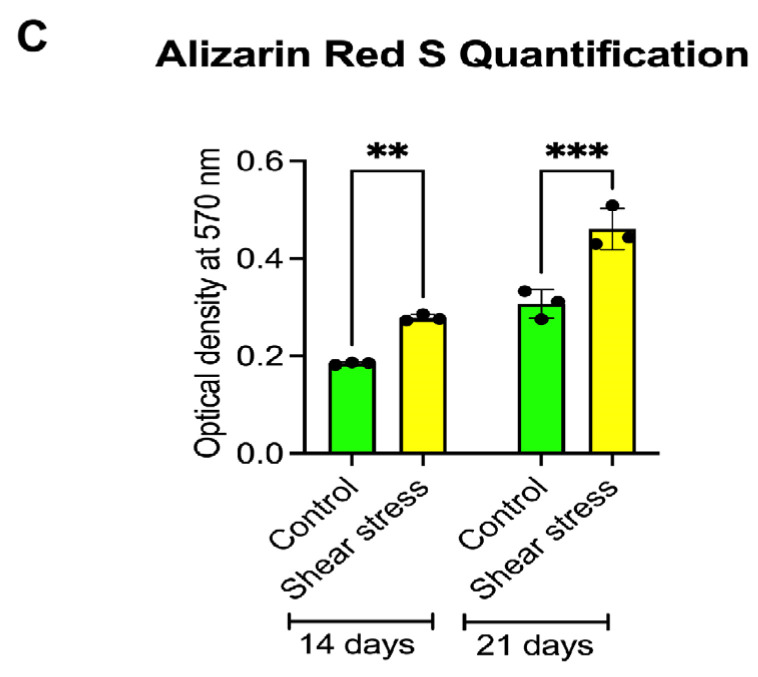
Shear stress promoted alkaline phosphatase activity and mineralization of hDPSCs. (**A**) Alkaline phosphatase staining of hDPSCs at Days 7, 14, and 21 of osteogenic induction. The magnification is 100×. The scale bars are 300 μm. (**B**) Alizarin Red S (ARS) staining revealed mineralization (red stain) after osteogenic differentiation for 14 and 21 days. The scale bar represents 300 μm. (**C**) Quantification of ARS staining was performed to determine calcification levels of Days 14 and 21 of hDPSCs undergoing osteogenic induction. Data were statistically analyzed by Mann–Whitney U test. (*n* = 3: different donors, *p* ** < 0.001, *** < 0.0001).

We investigated the influences of shear stress on the mineralization and calcification of hDPSCs using ALP staining and Alizarin Red S (ARS) staining on Days 7, 14, and 21 of osteogenic induction. ALP promotes the mineralization of inorganic phosphate by catalyzing the hydrolysis of organic phosphate esters, and serves as an early osteogenic marker [23]. Enhanced ALP staining was observed in the shear stress group on Days 14 and 21 (Figure 3A). In addition, ARS staining confirmed that the cell outgrowth areas displayed mineralization in the control static group, and the mineralization was further enhanced under the shear-stress loading group on Days 14 and 21 (Figure 3B). The quantification of ARS staining demonstrated that shear stress significantly enhanced mineralization of osteogenically induced hDPSCs (Figure 3C).

### 2.3. Identification of the Signaling Pathways Involved in Osteogenic Differentiation of hDPSCs After Shear-Stress Application

**Figure 4:**
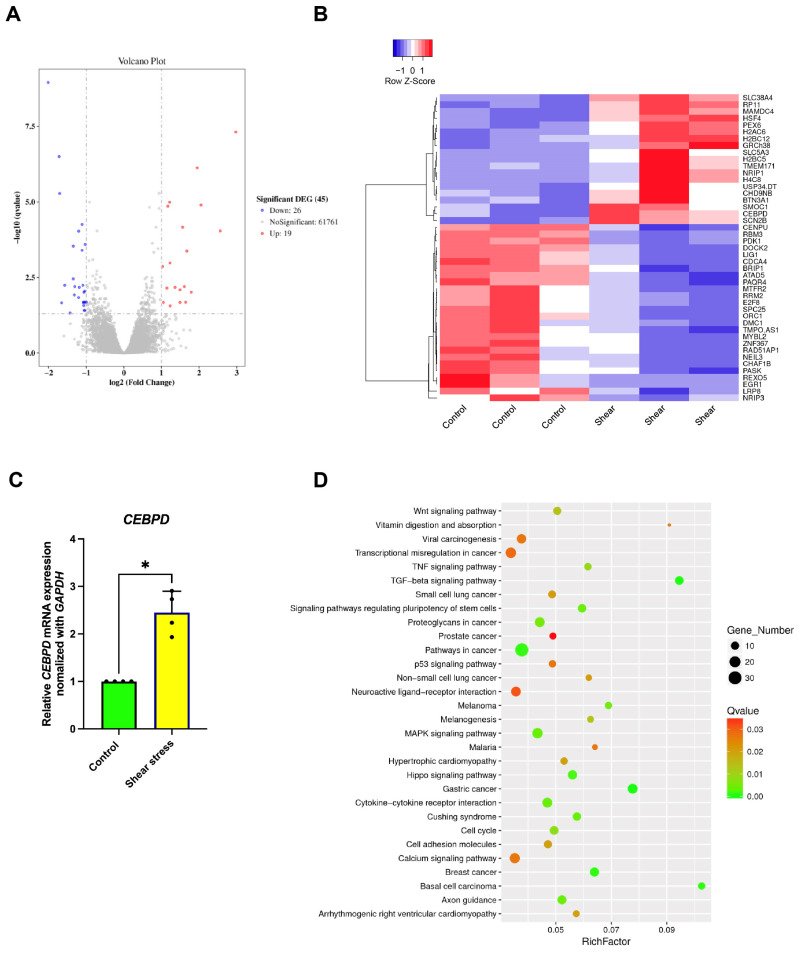
Identifying differential gene expressions (DEGs) of hDPSCs after applying shear stress for 24 h. (**A**) Volcano plot analysis for visualizing up-regulated (red) and down-regulated (blue) genes in the RNA sequencing dataset. (**B**) Heatmap showing differential gene expression profiles of hDPSCs after shear stress, comparing the control and shear stress groups. (**C**) Real-time RT-PCR was performed to detect mRNA expression levels of *CEBPD* gene. The expression of *GAPDH* was used as an internal control. Data were statistically analyzed by the Mann–Whitney U test. (*n* = 4: different donors, *p* * < 0.05). (**D**) Pathway enrichment of differentially expressed genes using the KEGG database showed the differentially regulated pathways after shear stress.

RNA sequencing data revealed an increase in gene expression potentially linked to the signaling pathways involved in the osteogenic differentiation of hDPSCs following the application of shear stress. Shear stress treatment resulted in 19 genes being upregulated and 26 genes being downregulated, as shown in the volcano plot (Figure 4A). A cluster heatmap shows differentially expressed genes resulting from shear stress compared to the control group. These genes were associated with biological processes, cellular components, and molecular functions. Among the genes which were upregulated following shear stress, the CCAAT enhancer-binding protein delta (*CEBPD*) was noted (Figure 4B). Real-time PCR analysis showed that shear stress significantly enhanced *CEBPD* expression (Figure 4C). Furthermore, KEGG pathway enrichment analysis showed an involvement of the MAPK signaling pathway in shear stress-treated hDPSCs. This included 11 upregulated and 7 downregulated differentially expressed genes (Figure 4D).

### 2.4. Shear Stress Stimulated Osteogenic Differentiation of hDPSCs Through p38 Mitogen-Activated Protein Kinase (MAPK) Pathway

**Figure 5:**
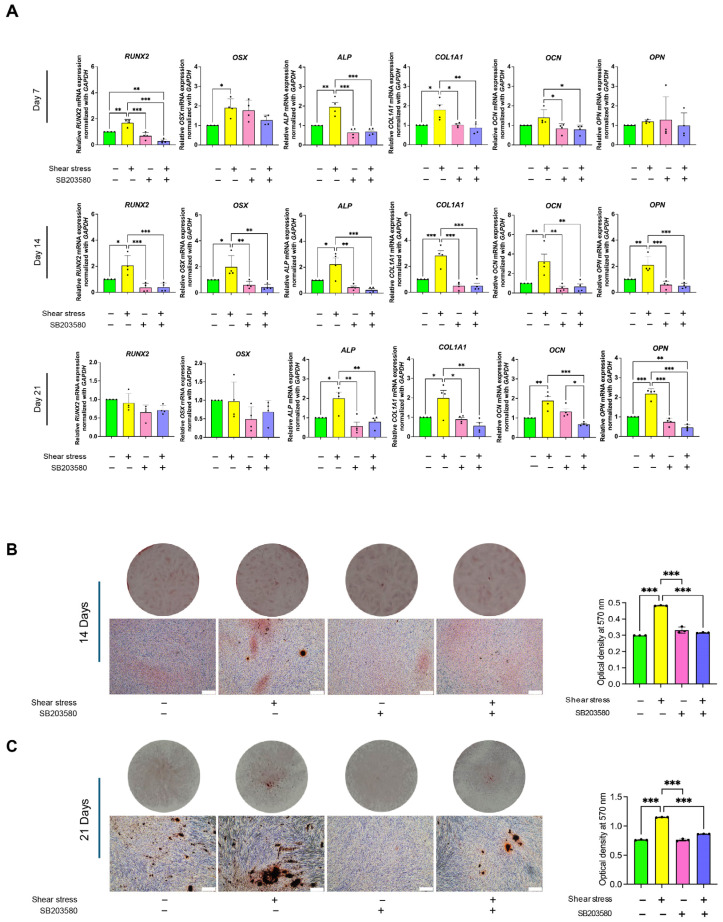
The p38 signaling pathway is associated with shear stress-induced osteogenic differentiation of hDPSCs. p38 inhibitor was applied to the cells prior to 24 h shear-stress application, then the cells were osteogenically induced for 21 days. The culture on Days 7, 14, and 21 was collected for real-time PCR analysis. The culture on Days 14 and 21 was collected for ARS staining. (**A**) Real-time RT-PCR was performed to detect mRNA expression levels of osteogenic marker genes, including RUNX2, OSX, ALP, COL1A1, OCN, and OPN. The expression of GAPDH was used as an internal control. Data were statistically analyzed by one-way ANOVA followed by Tukey’s multiple comparison tests. (*n* = 4: different donors, *p* * < 0.05, ** < 0.001, *** < 0.0001). ARS staining showed mineralization (red stain) and the right panel shows ARS quantification of osteogenically induced hDPSCs for 14 days (**B**) and 21 days (**C**). The scale bar represents 300 μm.

We identified the specific mechanism related to shear stress-induced osteogenic differentiation of hDPSCs. The hDPSCs were treated with p38 inhibitors (SB203580) before stress application. Then, after applying shear stress, the cells were maintained in osteogenic induction medium for 21 days. The mRNA expression of osteogenic marker genes, *RUNX2*, *OSX*, *ALP*, and *COL1A1*, was increased in the shear-stress loading group, while the p38 inhibition significantly blocked the upregulation of osteogenic marker genes for 14 days (Figure 5A). On day 21, shear stress upregulated osteogenic marker genes such as *ALP*, *COL1A1*, *OCN*, and *OPN*, while these upregulations were suppressed by p38 inhibitor treatment (Figure 5A). Additionally, the increased mineralization and calcification by shear-stress loading observed through ARS staining were significantly reduced in the p38 inhibitor treatment on Days 14 (Figure 5B) and 21 (Figure 5C).

## 3. Discussion

Stem cell-based tissue engineering is crucial for regenerative bone therapy, as it possesses osteoinductive properties. Dental stem cells, especially those sourced from dental pulp, periodontal ligaments, and alveolar bone, can differentiate into different cell types [24]. We previously demonstrated that our explant method provides dental pulp-derived stem cell isolation with mesenchymal characteristics [25,26]. The isolated cells from human dental pulp contain the mesenchymal cell surface markers, i.e., CD44, CD90, CD73, and CD105, and were negatively stained for hematopoietic cell marker CD45 [25,26]. In addition, the cells possessed multipotency, and could differentiate into osteoblasts, adipocytes, and so on [25,26]. Indeed, the isolated cells from human dental pulp by our culture system demonstrated the mesenchymal stem cell properties, which would be beneficial for stem cell-based tissue engineering. Although regenerative bone therapy has been studied for a long time, critical-sized defects still await effective strategies for the development of tissue-engineered bone.

Mechanical stimulation, such as shear force and compressive force, plays a crucial role in the function and behavior of dental stem cells, significantly influencing their regenerative abilities [27]. Previous reports have shown that different magnitudes of force, which can also be expressed in terms of stress, affect embryonic stem cell lineage commitment [28]. A specific shear stress of 0.5 Pa could induce endothelial differentiation of mouse embryonic stem cells [29,30]. Also, 0.5 Pa is the optimal stress to enhance the osteogenic differentiation of mouse induced pluripotent stem cells (iPSCs) [31]. A wide range of shear stress magnitudes has been demonstrated to influence mesenchymal stem cells’ behaviors [32,33], including their differentiation capacity toward specific cell types [34]. This study utilized a novel shear stress apparatus, with the procedures illustrated in Figure 1. Indeed, our demonstration showed that priming hDPSCs with shear stress could enhance osteogenic marker-gene expression of hDPSCs, along with osteogenic induction.

In bone tissue, the osteocytes lie in lacunae and are connected via dendritic processes projected in canaliculi. Osteocytes are the primary sensors of mechanical stimuli in bone, acting by sensing fluid shear, and can orchestrate osteoblast and osteoclast function to adapt bone structure and composition to meet physiological loading demands. The magnitude of the predicted fluid-induced shear stresses, 0.8 to 3 Pa, is shown to be similar to the fluid shear stresses measured in osteoblasts and other cells, in which an intracellular Ca2+ shear stress response had been observed [35]. This suggests that the 0.5 Pa of application is within the normal physiological range. In addition, our previous study was performed to evaluate the effects of shear stress on the osteogenic differentiation of iPSCs. It showed that the 0.5 Pa did not disturb the viability of the cells. Moreover, using this shear-stress loading apparatus, the viability of human periodontal ligament stem cells (hPDLSCs) was not influenced by 0.5 Pa shear-stress loading [32,33]. These could imply that 0.5 Pa is the optimal stress that does not affect the viability of hDPSCs.

Shear stress increased the mRNA expression of key osteogenic marker genes in both early and late stages of osteogenic differentiation of hDPSCs. The early markers of osteogenic commitment, i.e., *RUNX2*, *OSX*, *ALP*, *COL1A1*, were enhanced by shear stress on Day 7 (Figure 2A). The late markers related to bone extracellular matrix and mineralization, such as *OCN* and *OPN*, were increased by shear stress on Days 14 and 21 (Figure 2B,C), indicating the normal osteogenesis process of MSCs [36]. The master transcriptional regulators of early osteogenic differentiation are RUNX2 and OSX [37]. Runx2 expression was observed in non-skeletal tissues such as sperm and brain, while *Osx* knockout mice demonstrated complete abrogation of bone formation [38]. This suggests that OSX is a specific transcriptional factor for osteogenic differentiation. Indeed, the enhanced osteogenic differentiation of hDPSCs induced by shear loading was, in part, confirmed, based on the expression of OSX (Figure 2B–D)

The enhancement of osteogenic marker genes and proteins by shear-stress loading positively influences the mineralization process of osteogenically induced hDPSCs. The positive staining for ALP and ARS, which were further enhanced by shear-stress loading, (Figure 3A–C), indicated the enhanced mineralization of hDPSCs during both early and late stages of bone formation, consistent with earlier research [31,39]. These findings align with earlier research [31]. In addition, the present study exhibited the discrepancies of osteogenic potential of hDPSCs between different donors. The positive ARS staining of shear stress groups in Figure 5C showed lower osteogenicity of hDPSCs than in Figure 3B. Genetic and individual variations could affect the capacity of osteogenic potential in different donors [40]. Therefore, the increased expression of osteogenic markers and mineral deposition suggests a potential mechanism for utilizing shear stress to advance bone tissue engineering and regenerative dentistry efforts. Understanding the connection between mechanical forces and cellular reactions could lead to novel approaches for treating bone-related issues and enhancing results in tissue engineering.

The gene ontology categories that were enriched for the upregulated genes were associated with the cell cycle, while those for the downregulated genes pertained to small-molecule and cofactor metabolic processes. When analyzing the RNA sequences associated with gene expression in osteogenic signaling pathways, volcano plotting revealed that only 19 genes are upregulated, and 26 genes are downregulated, after shear stress treatment (Figure 4A). Over 60,000 differentially expressed genes are found to be insignificant. This may be concerned with the use of a single-end sequencing strategy in conducting RNA sequencing analysis. Single-end sequencing could lead to decreased accuracy in alignment and detection levels of structural features compared with paired-end sequencing. However, single-end sequencing shows expression gene analysis which is enough for a typical experimental design and also for simple data processing, and which is cost-effective [41]. A cluster heatmap demonstrated the list of differentially expressed genes after shear stress, compared to the control group (Figure 4B). Previous research showed that inhibition of the p38 MAPK signaling pathway led to decreased CEBPD and impaired osteogenesis [42]. This suggests that CEPBD may be involved in the regulation of osteogenesis via the p38 MAPK signaling pathway and is also associated with increased expression of *RUNX2* and *COL1A1* [43]. Our findings indicate that shear stress leads to an increase in *CEPBD* gene expression (Figure 4C). KEGG pathway enrichment analysis also indicated that the upregulated differentially expressed genes after shear stress were found in the MAPK signaling pathway among upregulated enriched pathways (Figure 4D).

The mitogen-activated protein kinase (MAPK) signaling pathway includes the family members of p38 MAPK, extracellular signal-regulated kinase 1/2 (ERK) and stress-activated protein (c-jun N-terminal, JNK) mitogen-activated signaling pathways. The KEGG database could not show which MAPK signaling pathway is associated with osteogenesis differentiation of hDPSCs. The previous study demonstrated that inhibition of p38 significantly decreased the ALP activity of osteoblasts treated with novel soy peptide (CBP), while ERK inhibitor and JNK inhibitor were not affected [44]. However, the ERK signaling pathway regulated the osteogenesis of human adipose-derived stem cells [45]. In contrast, activation of ERK negatively regulated the differentiation of osteoblast. Thus, consequently, we explored the effects of a p38 inhibitor to confirm whether the MAPK signaling pathway plays a role in regulating the osteogenic differentiation of hDPSCs following shear stress treatment.

**Figure 6:**
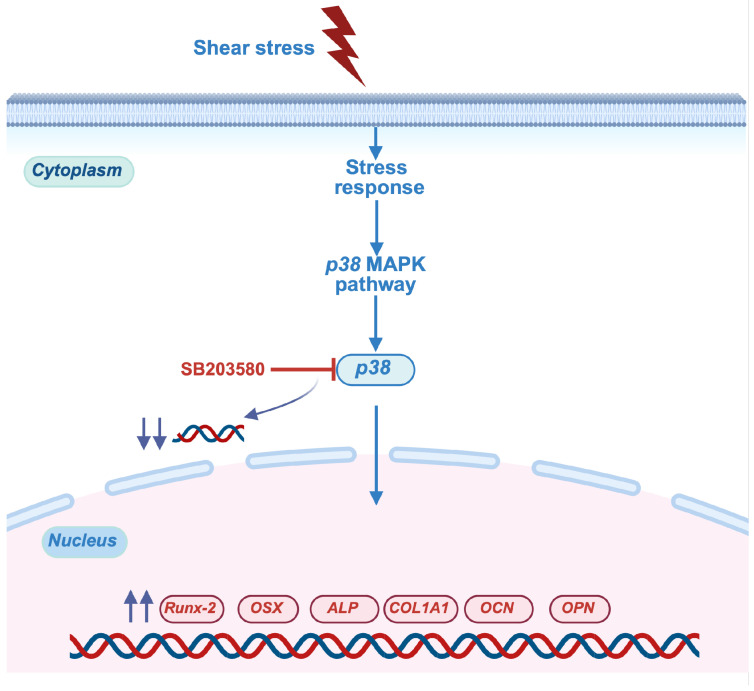
Schematic diagram demonstrating the involvement of p38 MAPK pathway as a part mechanism of the shear-stress-application-enhanced osteogenic differentiation of hDPSCs. This image was created in BioRender (version 04). Lwin, H. (2025) https://BioRender.com/mcrjdw8 (accessed on 10 June 2025).

Different mechanisms have been identified as involved in the induction of osteogenic differentiation of stem cells in response to mechanical stimuli, depending on the specific cell type and the magnitude of the mechanical loadings. Shear stress was found to enhance the osteogenic differentiation of mouse iPSCs through ERK1/2 signaling [31]. Intermittent compressive force facilitated the osteogenic differentiation of hPDLSCs through the transforming growth factor-beta (TGF-β) signaling pathway [39]. Our results demonstrated that most osteogenic marker-gene expression was completely abolished when the cellular response of hDPSCs was blocked by p38 inhibitor (Figure 5), but 2.5 μm of ERK inhibitor (ERKi) showed no effect on early and late osteogenic marker genes of shear stress-induced hDPSCs (Supplementary Figure S1). The ERK signaling pathway is not related with osteogenesis differentiation of hDPSCs, which is consistent with a previous study [46]. Our data suggested that the p38 MAPK signaling pathway might mediate the enhancing effect of shear stress on osteogenic differentiation of hDPSCs, indicating that shear stress could regulate the osteogenic differentiation of hDPSCs through the p38 MAPK pathway. Figure 6 illustrates the proposed mechanism by which shear stress influences the osteogenic differentiation of hDPSCs.

The MAPK signaling pathway plays a significant role in cell proliferation and differentiation, and also affects the osteogenic differentiation of MSCs. The previous study proved that activation of the MAPK signaling pathway led to osteogenic differentiation of pre-osteoblasts, the MC3T3-E1 cells [43]. The MAPK signaling pathway also plays a critical role in regulating osteogenic differentiation of PDLSCs [47]. However, further understanding of MAPK signaling in the regulation of osteogenic differentiation of hDPSCs is still unclear. Osteoblast differentiation relies on the coordinated interaction of various cell signaling pathways. Various signaling pathways, including TGF-β/ bone morphogenetic proteins, Notch, Hedgehog, Wnt/β-catenin, and MAPK, induce osteogenic lineage commitment and differentiation of osteoblasts [48,49,50,51]. All these pathways work together to regulate the osteogenic differentiation processes that ultimately lead to bone formation [52]. Among these signaling pathways, p38 MAPK signaling is a highly conserved pathway essential for several developmental processes, including bone formation and regeneration [53].

Fluid shear stress influences osteoblast cell behaviors, that is, differentiation and maturation, depending on the stress magnitude and application duration. Shear stress has been reported to promote the osteogenic differentiation and maturation of osteoblastic cells derived from MSCs [54,55]. High-force magnitudes have been reported to induce nitric oxide, adenosine triphosphate, prostaglandin E2, and OPN production by pre-osteoblastic cells [56] and osteocytes [57]. Those molecules appear to influence the inflammatory process [58] and angiogenesis [59]. Controlling angiogenesis and optimal inflammatory responses are crucial for regenerative bone therapy [60,61].

Our results indicate that shear stress significantly enhances the osteogenic differentiation of hDPSCs. However, hDPSCs also have the ability to differentiate into multiple lineages, including chondrogenic and adipogenic pathways [62,63]. This study specifically focuses on osteogenic differentiation; therefore, additional studies are needed to find out whether this shear stress affects the pathways involved in chondrogenic and adipogenic differentiation of hDPSCs. Previous studies have showed that shear stress can influence the differentiation of different cell types; for instance, fluid flow-induced shear stress stimulates bone marrow-derived MSCs into the osteoblastic phenotype, without additional chemical induction [64]. Furthermore, fluid shear stress suppresses the maturation of adipocytes [65]. Responses to shear stress differ based on the cell type, and understanding how different cells react to shear forces could inform the creation of targeted treatment strategies that leverage the mechanosensitivity of various types of MSCs.

Translation of shear stress from a laboratory setting to a clinical environment represents a promising opportunity for enhancing tissue regenerative therapies; however, the feasibility of this clinical potential relies on the ability to create dynamic mechanical environments that accurately mimic physiological conditions in vivo. Consequently, comprehensive preclinical studies are required to optimize several parameters, like the intensity, frequency, and duration of shear stress, which are required to induce the intended differentiation pathways in hDPSCs. Furthermore, regulatory frameworks for bioreactors and stem cell therapies need careful guideline to ensure safety and evaluate the long-term effects of these treatments in different clinical situations.

Although a two-dimensional (2D) model is helpful for understanding the basic cellular response to shear stress, it has significant limitations; the lack of the complete architecture and interactions found in a three-dimensional (3D) environment can result in inaccurate mechanostransduction representations. Additionally, a 2D model is inadequate for better mimicking the complex cellular interactions and ECM components that are crucial in the differentiation of hDPSCs, suggesting that findings from additional studies require significant validation through a 3D model before clinical application.

Various physical forces, such as compression, tension, and vibration, play a crucial role in the osteogenic differentiation of hDPSCs. For instance, both direct and hydrostatic compression promote osteogenic differentiation by increasing NOTCH2 expression [66]. In contrast, uniaxial tensile stretch suppresses the osteogenic differentiation by inhibiting BMP2, OCN, and ALP expression by hDPSCs [67]. Additionally, medium-magnitude sonic vibration promotes osteogenic differentiation by inducing the G0/G1 arrest of hDPSCs via the *p*-ERK/Runx-2 pathway [10]. These findings highlight the various mechanical forces that participate in the regulation of osteogenic differentiation of hDPSCs, enhancing our understanding of how multifaceted mechanical forces contribute to the complex process of osteogenic differentiation of hDPSCs and potentially improving the therapeutic potential of these mechanical settings in stem cell-based regenerative treatments.

Although the p38 MAPK signaling pathway plays a significant role in osteogenesis, its role is complex and has several limitations that may affect its potential as a therapeutic target. Along with c-Jun N-terminal kinase, p38 MAPK acts as stress-activated protein kinases through a wide range of environmental stresses, such as the production of cytokines, inflammatory reactions, the cell cycle, and differentiation and apoptosis. While inflammation is part of bone remodeling, chronic inflammation can impair osteogenesis and contribute to diseases like osteoporosis and rheumatoid arthritis [68]. In addition, p38 MAPK signaling enhances the effects of RANKL on the induction of osteoclast differentiation, and osteoclast-mediated bone resorption in myeloma cells triggers osteolytic bone destruction [69]. p38 MAPK in breast cancer cells plays an important role in osteoclast differentiation and activity [70]. Systemic inhibition of p38 can have off-target effects, impairing immune function or tissue repair [71]. Selective p38 inhibitors have been developed, but show limited clinical success, due to poor specificity.

While our research demonstrated that shear stress enhanced the osteogenic differentiation of hDPSCs, further studies are needed to explore the specific impact of shear stress on different aspects, i.e., inflammation and angiogenesis, of osteogenically induced hDPSCs on the surrounding cells. Overall, shear stress could be suggested as a promising factor for enhancing in vitro tissue-engineered bones intended for regenerative bone therapy. Consequently, hDPSCs exposed to shear loading could improve technological advancements in stem-cell-based regenerative therapies in dentistry, serving as a promising method for restoring bone height and width for prosthetic or implant procedures.

## 4. Materials and Methods

### 4.1. Cell Culture

hDPSCs were collected individually from third molars of patients aged between 20 and 30 years who came to the Department of Oral and Maxillofacial Surgery, Faculty of Dentistry, Chulalongkorn University. The teeth were extracted for impaction or orthodontic reasons. Written consent was obtained from each patient, and the protocol was approved by the Human Research Ethical Committee of the Faculty of Dentistry, Chulalongkorn University (approval no. HRE-DCU 2023-002) and conducted under the guidelines of the Biosafety Committee of the Faculty of Dentistry, Chulalongkorn University. In brief, dental pulp tissues were collected, minced, and placed on 35 mm tissue culture dishes for 3–4 weeks, to obtain cell outgrowth. The growth medium consisted of Dulbecco’s Modified Eagle Medium (DMEM, Gibco, Waltham, MA, USA) enriched with 10% fetal bovine serum (FBS, Gibco, Waltham, MA, USA), 2 mM L-glutamine (GlutaMAX-1, Gibco, Waltham, MA, USA), and antibiotics including 100 units/mL penicillin, 100 μg/mL streptomycin, and 250 ng/mL amphotericin B (Antibiotic–Antimycotic, Gibco, Waltham, MA, USA). The cells were incubated at 37 °C in a humidified atmosphere of 5% carbon dioxide. The culture medium was changed every 2 days. Cells were subcultured at a 1:3 ratio using 0.25% trypsin EDTA (Gibco, Waltham, MA, USA) once they reached 80% confluency, and subsequent experiments were conducted with cells between passages 3 and 7.

### 4.2. Shear-Stress Application

The hDPSCs at a cell density of 3 × 10^5^ cells/cm^2^ were seeded in 35 mm dish overnight. The growth medium was changed to 4 mL per dish prior to 0.5 Pascal (Pa) shear-stress application, for 24 h [31]. The control group was not subjected to the shear stress, which is so-called static culture. After applying shear stress, the hDPSCs were maintained in osteogenic induction medium (OM) for 21 days. The medium was changed every 2 days. The OM consisted of Dulbecco’s Modified Eagle Medium (DMEM, Gibco, Waltham, MA, USA) supplemented with 10% fetal bovine serum (FBS, Gibco, Waltham, MA, USA), 2 mM L-glutamine (GlutaMAX-1, Gibco, Waltham, MA, USA), 100 unit/mL penicillin, 100 μg/mL streptomycin, 250 ng/mL amphotericin B (Antibiotic–Antimycotic, Gibco, Waltham, MA, USA), 50 μg/mL ascorbate-2-phosphate (Sigma-Aldrich, St. Louis, MO, USA), 100 nm dexamethasone (Sigma-Aldrich, St. Lousi, MO, USA), and 5 mM β-glycerophosphate (Sigma-Aldrich, St. Lousi, MO, USA). The detailed procedures are shown in Figure 1.

### 4.3. Real-Time Polymerase Chain Reaction (PCR) Analysis

Total cellular RNA was extracted using RiboEx^TM^ solution. RNA was converted into complementary DNA using a reverse transcription kit (complementary DNA using a reverse transcription kit, ImProm-II Reverse Transcription System, Promega, Madison, WI, USA) and subjected to real-time PCR analysis. Cycling conditions were set at 95 °C for 30 s followed by 40 cycles of 95 °C for 3 s and 60 °C for 30 s. Real-time RT-PCR using the Green Master Mix (Promega Corporation, Madison, WI, USA) was performed on a Roche real-time PCR system, according to the manufacturer’s instructions. Relative gene expression was quantified by the 2^−ΔΔCT^ method and normalized to the expression of the glyceraldehyde 3-phosphate dehydrogenase gene. The early and late osteogenic markers such as Runt-related transcription factor 2 (*RUNX2*), osterix (*OSX*), alkaline phosphatase (*ALP*), Type I collagen (*COL1A1*), osteocalcin (*OCN*), osteopontin (*OPN*), and CCAAT enhancer-binding protein delta (*CEBPD*) were used in the study. The primer sequences used for real-time RT-PCR are listed in Table 1.

### 4.4. Alkaline Phosphatase (ALP) Staining

After being incubated for all time periods, the cells were washed twice with phosphate buffer saline (PBS, Boston, MA, USA) and subsequently fixed with 4% paraformaldehyde for 15 min. A solution of 180 mM of Fast Red TR salt (Sigma, St. Lousi, MO, USA) in distilled water and a 90 mM Naphthol AS-MX solution in N, N-dimethylformamide were prepared separately, then mixed with 120 mM Tris (hydroxymethyl) aminomethane (Sigma-Aldrich, St. Lousi, MO, USA) buffer. The cells were stained with this mixture and incubated at 37 °C for 20 min. Following adequate incubation for cell staining, cells were washed with PBS and dried overnight. Digital images were captured using a light microscope.

### 4.5. Alizarin Red S (ARS) Staining and Quantification

ARS staining was accessed on Days 14 and 21 of the incubation periods. Prior to fixation with 4% paraformaldehyde in phosphate buffer, the cells were washed with PBS. The cells were then incubated in 2% ARS (Sigma, St. Lousi, MO, USA) solution with a pH range from 4.1 to 4.3 for 20 min, washed with distilled water, and allowed to dry before capturing digital images. The images were taken under a light microscope.

The mineral deposits were dissolved with 10% cetylpyridinium chloride monohydrate (Sigma-Aldrich, St. Lousi, MO, USA) in phosphate buffer, for quantitative analysis. The solution was transferred to a 96-well plate and the optical density was measured at 570 nm with a microplate reader.

### 4.6. Immunofluorescence Staining

hDPSCs were seeded in a growth medium at a cell density of 3 × 10^5^ cells/cm^2^ on coverslips that had been placed in a 35 mm dish, allowing for overnight incubation. After 24 h of shear-stress application, the hDPSCs were maintained in an OM for 14 days. The cells were then fixed using 4% paraformaldehyde for 15 min and permeabilized with 0.1% Triton X-100. After gently washing with PBS for 2 times, non-specific binding was blocked using 3% bovine serum albumin (BSA) (Capricorn Scientific, Ebsdorfergrund, Germany) blocking buffer for 1 h. The samples were subsequently incubated overnight at 4 °C with a primary antibody: anti-osterix monoclonal antibody (F-3, sc-393325: 1:50 dilution in 1% BSA, Santa Cruz Biotechnology, Dallas, TX, USA). After overnight incubation, the samples were incubated with an Alexa Fluor 488-conjugated goat anti-mouse IgG (1:1000 dilution in 1% BSA, Molecular Probes, Thermo Fisher Scientific, Waltham, MA, USA) for 1 h at room temperature. The samples were also incubated with Rhodamine-phalloidin (1:1000 dilution in 1% BSA, Cytoskeleton, Denver, CO, USA), using PBS as the diluent. The samples were then washed with PBS and mounted onto slides with an anti-fade mounting medium containing DAPI (Vectashield, Vecta Laboratory, Dallas, TX, USA). Immunofluorescent microscopy and analysis were performed under an apotome fluorescent microscope (Axio Observer Z1 and ZEN pro, ZEISS International, Oberkochen, Germany).

### 4.7. Western Blotting Analysis

After 14 days of osteogenic induction, the samples were washed with PBS and lysed using ultrasonic homogenization in RIPA buffer (Wako Pure Chemical, Richmond, VA, USA) supplemented with a protease inhibitor (Cytoskeleton, Denver, CO, USA). To measure the protein concentration in each sample, the Pierce BCA protein assay was performed, according to the manufacturer’s instruction (Thermo Fisher Scientific, Waltham, MA, USA). Protein samples (30 μg) were loaded onto a 12% polyacrylamide gel (SDS-PAGE) for electrophoresis and then transferred onto a polyvinylidene fluoride membrane (Bio-Rad Laboratories, Hercules, CA, USA). The membranes were blocked using 5% BSA in TBST (10 mM Tris–HCl, pH 7.4, 100 mM NaCl, and 0.1% Tween), and then incubated with anti-OSX monoclonal antibody (F-3, sc-393325: 1:1000 dilution in 3% BSA, Santa Cruz Biotechnology, Dallas, TX, USA) at 4°C overnight or anti-GAPDH (6C5, 1:5000 dilution in 1% skim milk, Millipore) for 3 h, at room temperature. After washing with TBST, the membranes were treated with a horseradish peroxidase (HRP)-conjugated anti-mouse IgG secondary antibody (abcam7076, 1:5000 dilution in 3% BSA or 1% skim milk, respectively, Santa Cruz Biotechnology, Dallas, TX, USA) at room temperature, for 1 h. Signal detection and quantitative analysis of relative protein expression were performed using chemiluminescence image analyzer (GE Healthcare, Pittsburgh, PA, USA) and ImageJ software (version 1.54g) (National Institutes of Health, Bethesda, MD, USA), respectively.

### 4.8. RNA Sequencing

Total cellular RNA was extracted using Ribospin II (GeneAll, Seoul, Republic of Korea) after being stimulated with shear stress for 24 h. The quality and quantity of RNA were initially measured using Nanodrop and further assessed with an Agilent 2100 BioAnalyzer (Agilent Technologies, Santa Clara, CA, USA), as well as a Qubit RNA HS assay kit (Thermo Fisher Scientific, Waltham, MA, USA). Single-ended sequencing was conducted on an Illumina Nextseq platform with 75 cycles. Differential gene expression was analyzed using DEseq2, with a statistically significant difference defined as a false discovery rate of less than 0.05. A heatmap was generated using Heatmapper.

### 4.9. Inhibition of p38 MAPK Pathway

hDPSCs were pretreated with p38 inhibitor (SB203580) at a concentration of 15 nm [72] or 25 μm of ERK inhibitor (ERKi) [73] for 30 min, prior to shear-stress application. After changing the medium, the cells were subjected to shear stress for 24 h. Subsequently, the cells were incubated in OM for 21 days. The OM was changed every 2 days.

### 4.10. Statistical Analysis

Statistical analyses were performed using GraphPad Prism software version 9.0 for Mac (GraphPad Software, San Diego, CA, USA). The normal distribution of data was analyzed by the Shapiro–Wilk test. Between-group differences were determined using unpaired t test followed by nonparametric Mann–Whitney U test. For more than 2 groups, differences were determined by parametric One-way ANOVA followed by Tukey’s multiple comparison tests. The *p* values of <0.05 were considered significant.

## 5. Conclusions

Shear stress can enhance the osteogenic differentiation of hDPSCs through the p38 MAPK signaling pathway (Figure 6). The findings from these studies will expand our understanding of tissue engineering and regenerative medicine, potentially leading to innovative approaches for improving bone repair and regeneration.

## Figures and Tables

**Table 1:** The sequences of primers used in the study.

Gene		Sequence
*RUNX2*	Forward	ATGATGACACTGCCACCTCTGA
	Reverse	GGCTGGATAGTGCATTCGTG
*OSX*	Forward	GCCAGAAGCTGTGAAACCTC
	Reverse	GCTGCAAGCTCTCCATAACC
*ALP*	Forward	CGAGATACAAGCACTCCCACTTC
	Reverse	CTGTTCAGCTCGTACTGCATCATGTC
*COL1A1*	Forward	GTGCTAAAGGTGCCAATGGT
	Reverse	ACCAGGTTCACCGCTGTTAC
*OCN*	Forward	CTTTGTGTCCAAGCAGGAGG
	Reverse	CTGAAAGCCGATGTGGTCAG
*OPN*	Forward	AGGAGGAGGCAGAGCACA
	Reverse	CTGGTATGGCACAGGTGATG
*CEBPD*	Forward	AGCGCAACAACATCGCCGTG
	Reverse	GTCGGGTCTGAGGTATGGGTC
*GAPDH*	Forward	CACTGCCAACGTGTCAGTGGTG
	Reverse	GTAGCCCAGGATGCCCTTGAG

## Data Availability

The datasets used and/or analyzed during the current study are available from the corresponding author upon reasonable request.

## Supplementary Materials

The following supporting information can be downloaded at: https://www.mdpi.com/article/10.3390/ijms26125667/s1.
