# Supplementary material for: Shear Stress Regulates Osteogenic Differentiation of Human Dental Pulp Stem Cells via the p38 Pathway
Source: Int J Mol Sci. 2025 Jun 13;26(12):5667. doi: 10.3390/ijms26125667 (PMC12193168; doi:10.3390/ijms26125667)
Supplement: Supplementary file 1 [file ijms-26-05667-s001.zip › ijms-3685378-supplementary.pdf]

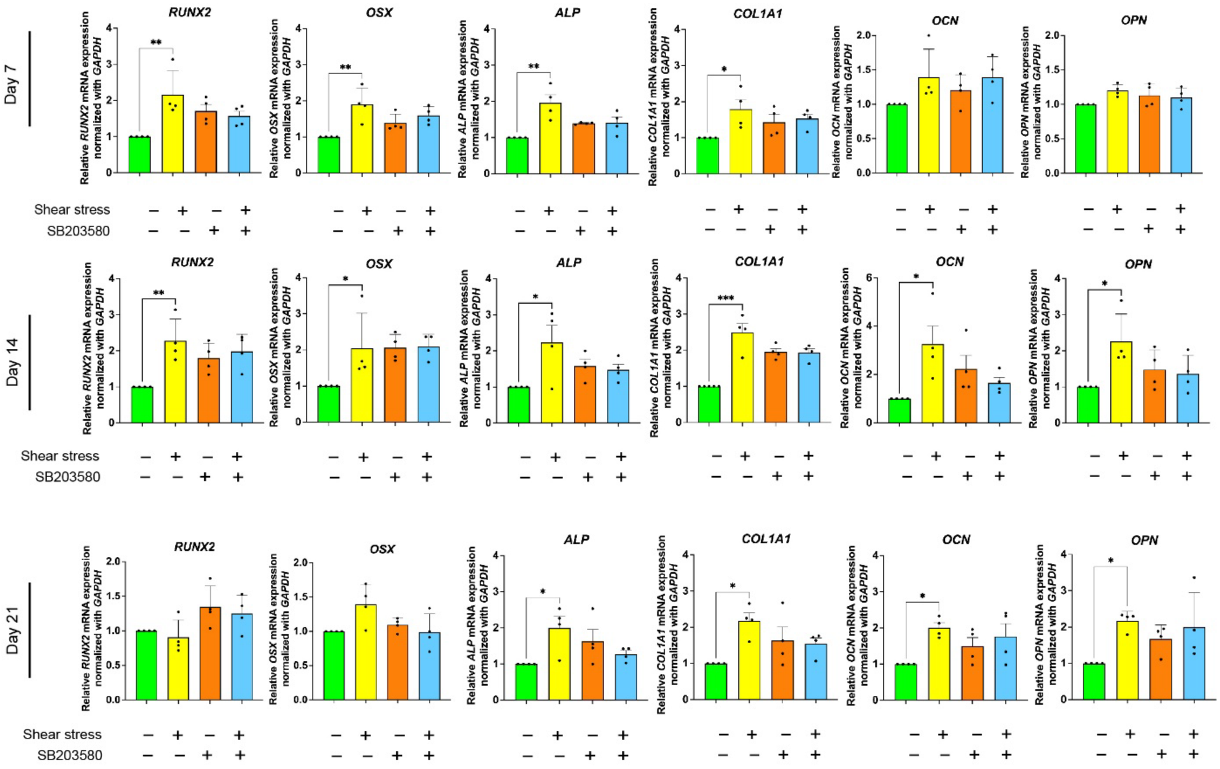

Supplementary Figure 1. The inhibition of ERK signaling pathway is not related with shear stress-induced osteogenic differentiation of hDPSCs. ERK inhibitor (ERKi) was applied to the cells prior to 24-hour shear stress application, then the cells were osteogenically induced for 21 days. The culture on Days 7, 14, and 21 was collected for real-time PCR analysis. Real-time RT-PCR was performed to detect mRNA expression levels of osteogenic marker genes, including *RUNX2*, *OSX*, *ALP*, *COL1A1*, *OCN*, and *OPN*. The expression of *GAPDH* was used as an internal control. Data were statistically analyzed by one-way ANOVA followed by Tukey's multiple comparison tests. (n=4, different donors, P *<0.05, **<0.001, ***<0.0001)
